# Emergence of a Multidrug-Resistant Hypervirulent Klebsiella pneumoniae Sequence Type 23 Strain with a Rare *bla*_CTX-M-24_-Harboring Virulence Plasmid

**DOI:** 10.1128/AAC.02273-18

**Published:** 2019-02-26

**Authors:** Dingxia Shen, Guannan Ma, Cuidan Li, Xinmiao Jia, Chuan Qin, Tingting Yang, Lifeng Wang, Xiaoyuan Jiang, Nan Ding, Xiuli Zhang, Liya Yue, Zhe Yin, Lijun Zeng, Yongliang Zhao, Dongsheng Zhou, Fei Chen

**Affiliations:** a Center of Clinical Laboratory Medicine, Chinese General Hospital of PLA, Beijing, China; b CAS Key Laboratory of Genome Sciences & Information, Beijing Institute of Genomics Chinese Academy of Sciences, Beijing, China; c University of Chinese Academy of Sciences, Beijing, China; d Central Research Laboratory, Peking Union Medical College Hospital, Peking Union Medical College & Chinese Academy of Medical Sciences, Beijing, China; e State Key Laboratory of Pathogen and Biosecurity, Beijing Institute of Microbiology and Epidemiology, Beijing, China; f CAS Key Laboratory of Genomic and Precision Medicine, Beijing Institute of Genomics, Chinese Academy of Sciences, Beijing, China

**Keywords:** *Klebsiella pneumoniae*, ST23, *bla*_CTX-M-24_, high-pathogenicity island, horizontal gene transfer (HGT), mobile genetic elements (MGEs), multidrug-resistant and hypervirulent *Klebsiella pneumoniae* (MDR-HvKP), virulent plasmid

## Abstract

Here, we report a multidrug-resistant hypervirulent Klebsiella pneumoniae (MDR-HvKP) strain of sequence type 23 (ST23) with a rare hybrid plasmid harboring virulence genes and *bla*_CTX-M-24_, and we analyze the genetic basis for relationship between genotypes and MDR-hypervirulence phenotypes. Further analysis indicates that the hybrid plasmid is formed by IS*903D*-mediated intermolecular transposition of the *bla*_CTX-M-24_ gene into the virulence plasmid. The emergence of MDR-HvKP strains, especially those carrying drug-resistant virulent plasmids, poses unprecedented threats/challenges to public health. This is a dangerous trend and should be closely monitored.

## Abstract (precis)

Here, we report a multidrug-resistant hypervirulent Klebsiella pneumoniae (MDR-HvKP) strain of sequence type 23 (ST23) with a rare hybrid plasmid harboring virulence genes and *bla*_CTX-M-24_, and we analyze the genetic basis for relationship between genotypes and MDR-hypervirulence phenotypes. Further analysis indicates that the hybrid plasmid is formed by IS*903D*-mediated intermolecular transposition of the *bla*_CTX-M-24_ gene into the virulence plasmid.

## INTRODUCTION

Hypervirulent Klebsiella pneumoniae (HvKP) is a serious threat to public health, as it could cause severe infection with high mortality and morbidity in young/healthy individuals (1). Previous studies showed that most HvKP strains are sensitive to common antibiotics, whereas multidrug-resistant (MDR) strains possess lower virulence (2). In addition to the differentiated phenotypes, HvKP and MDR K. pneumoniae show different genotypes with different clonal groups (3). However, recent studies found that several HvKP and MDR strains could evolve into MDR-HvKP strains through acquiring multidrug-resistant or hypervirulent plasmids (4–7). The emergence and spread of MDR-HvKP is undoubtedly one of the most severe threat/challenges to global public health. Understanding of the genetic basis for MDR-HvKP strains is essential to control this deadly infection. In this study, we reported an MDR-HvKP strain of sequence type 23 (ST23) with a rare plasmid harboring virulence and *bla*_CTX-M-24_ genes, and we analyzed the genetic basis for relationship between genotypes and MDR-hypervirulence phenotypes.

MDR-HvKP strain 11492 was isolated from the blood of a male community infection patient (in his 30s) with an abscess of the kidney at the Chinese PLA General Hospital in November 2014. The patient was diagnosed with pancreatitis, pancreatic abscess, high fever (∼40.8°C), and shock (for detailed clinical information, see Table S1). The patient was subjected to antibiotic treatment (including imipenem-cilastatin sodium, teicoplanin, linezolid, meropenem, and biapenem), and operated on to remove the abscess.

Serotyping and multilocus sequence typing (MLST) analyses indicated that strain 11492 belong to serotype K1 and ST23. The MDR-HvKP phenotype of strain 11492 was then characterized by string test, Galleria mellonella infection test, and drug susceptibility test (described below). An MDR-KP strain, 13190 (ST392/K27), and an HvKP strain, NTUH-K2044 (ST23/K1), were also included as controls (8, 9).

**FIG 1:**
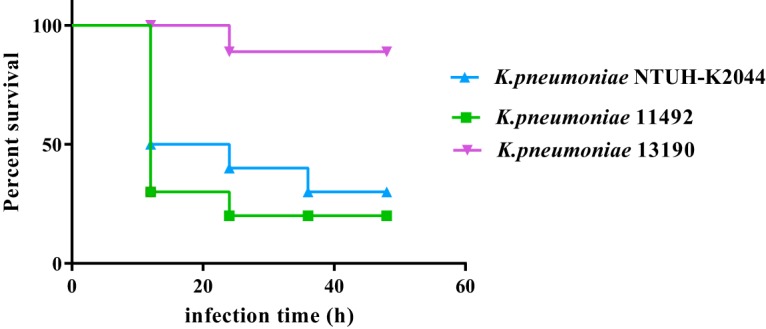
Virulence phenotype characterization of three K. pneumoniae strains using a G. mellonella infection model. The virulence was determined by the survival rates of G. mellonella larvae infected with the strains (1 × 10^6^ CFU/ml).

We first evaluated the virulence phenotype of the strains using a string test. Strains 11492 and NTUH-K2044 showed a hypermucoviscous phenotype (viscous strings of >5 mm). We further analyzed the hypervirulent phenotype by wax moth larvae (Galleria mellonella; Huiyude Biotech Company, Tianjin) infection testing. G. mellonella larvae were maintained on wood chips in the dark at 15°C until use. We then infected the G. mellonella larvae with 10^4^, 10^5^, 10^6^, and 10^7^ CFU/ml of K. pneumoniae strains. Experiments were performed in triplicate. The virulence of K. pneumoniae was determined by the survival rate of the G. mellonella larvae, using GraphPad Prism software 5.01 (10, 11). The result indicated a significant decrease in survival rates when infected with 11492 and NTUH-K2044 HvKP strains in relative to those when infected with classical K. pneumoniae (cKP) strain 13190 under various infection concentrations at multiple time points (Fig. 1, Fig. S1 and S2). Thus, using HvKP strain NTUH-K2044 as the hypervirulence-positive control and cKP strain 13190 as the negative control, strain 11492 was demonstrated to be hypervirulent.

Drug susceptibility testing was performed by broth microdilution using 23 antibiotics, and the drug resistance phenotypes were determined according to CLSI standards (12). Strain 11492 was resistant to 10 antibiotics, including penicillin (ampicillin [AMP], MIC ≥ 512 mg/liter), cephalosporin I (cefazolin [CFZ], MIC ≥ 256 mg/liter), cephalosporin II (cefuroxime [CXM], MIC ≥ 512 mg/liter), cephalosporin III (ceftazidime [CAZ], MIC ≥ 256 mg/liter), cephalosporin IV (cefipime [FEP], MIC ≥ 128 mg/liter), macrolides (erythromycin [ERY], MIC ≥ 64 mg/liter; azithromycin [AZM], ≥ 32 mg/liter), tetracycline (tetracycline [TET], MIC ≥ 32 mg/liter), amphenicol (chloramphenicol [CHL], MIC ≥ 32 mg/liter), and monobactams (aztreonam [ATM], MIC ≥ 16 mg/liter), indicating an MDR phenotype. It was also intermediately resistant to 2 antibiotics, including cephamycin (cefoxitin [FOX], MIC = 16 mg/liter) and nitrofuran (nitrofurantoin [NIT], MIC = 64 mg/liter). On the other hand, it was sensitive to 11 antibiotics, including carbapenems (imipenem [IMP], MIC ≤ 1 mg/liter; meropenem [EME], MIC ≤ 1 mg/liter), aminoglycosides (gentamicin [GEN], MIC ≤ 4 mg/liter; amikacin [AMK], MIC ≤ 8 mg/liter), fluoroquinolones (ciprofloxacin [CIP], MIC ≤ 1 mg/liter; levofloxacin [LVX], MIC ≤ 2 mg/liter), tetracycline (minocycline [MIN], MIC ≤ 1 mg/liter), fosfomycin (fosfomycin [FOF], MIC ≤ 64 mg/liter), sulfanilamide (trimethoprim-sulfamethoxazole [TMP-SMX], MIC ≤ 1/19 mg/liter), glycylcycline (tigecycline [TGC], MIC ≤ 1 mg/liter), and glycopeptide (polymyxin E [PE], MIC ≤ 1 mg/liter).

To reveal the genetic basis of the MDR-hypervirulence phenotype, we obtained the complete genome of MDR-HvKP 11492 by single-molecule real-time (SMRT) sequencing (Table S2 and Fig. S3) (GenBank accession no. CP026021 to CP026022). *De novo* assembly was performed using the Hierarchical Genome Assembly Process 3 (HGAP3) within SMRT Analysis v2.3.0. Gap closing was completed by PBJelly (13), and circularization was achieved by manual comparison. To correct the polymer errors, we resequenced these isolates using Illumina sequencing (Table S3). Raw reads were trimmed, filtered, and mapped onto assembled genome sequences using BWA v0.5.9. Pilon v1.13 was subsequently employed to polish genome sequences using the obtained alignments (14). Genome sequences were annotated with the Rapid Annotations using Subsystem Technology (RAST) pipeline (15).

Bioinformatic analysis provided the general chromosome genome information (Table S4), including GC% content (57.45%), genome size (5.25 Mb), predicted protein-coding genes (5,017), gene length (922 bp), and coding region (88.1%). Strain 11492 contained a 193-kb plasmid, p11492-vir-CTXM, with a lower GC content (50.37%), lower ratio of coding regions (75.71%), and shorter average gene length (724 bp).

**FIG 2:**
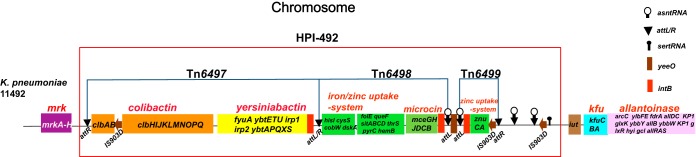
Genetic structure of virulence genes clusters on the chromosome and related HPI (HPI-492). The different color-coded modules represent different virulence gene clusters. The regions in red boxes denotes HPI-492. The *attL* and *attR* (*attL/R*) sequences, *asn* tRNA and *ser* tRNA genes are marked in the regions.

**FIG 3:**
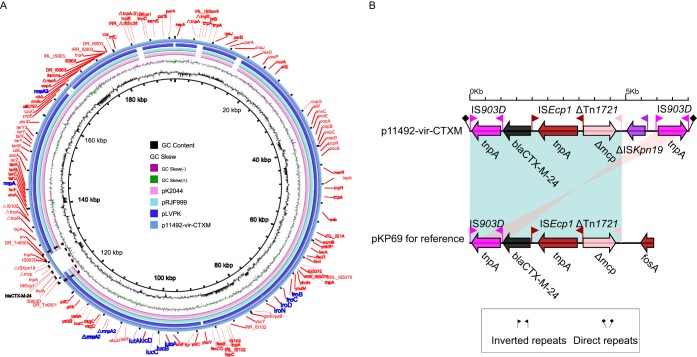
Structure of the hybrid drug-resistant virulent plasmid p11492-vir-CTXM and corresponding novel transposon Tn*6501* with *bla*_CTX-M-24_. (A) Structural comparison of the hybrid drug-resistant virulent plasmid p11492-vir-CTXM and three reference virulence plasmids. Alignment of plasmid pRJF999, pK2044, pLVKP, and p11492-vir-CTXM are shown as concentric rings. The outermost show the main coding genes of p11492-vir-CTXM. Virulence genes are highlighted in blue. Resistant gene *bla*_CTX-M-24_ is shown in black. (B) Structural comparison of Tn*6501* with a drug-resistant gene region (*bla*_CTX-M-24_) in p11492-vir-CTXM and the *bla*_CTX-M-24_-embedded region of plasmid pKP69.

Virulence genes were identified by BLAST, based on the database from Pasteur Institute (95% coverage and 95% identity cutoff). Strain 11492 has 71 virulence genes belonging to nine virulence gene clusters, which contains almost all of the identified 85 virulence genes of K. pneumoniae strains (16). Seven clusters are located on the chromosome (*mrk*, yersiniabactin, allantoinase, colibactin, iron/zinc acquisition-system, *kfu*, and microcin). Four virulence gene clusters (colibactin, yersiniabactin, iron/zinc acquisition-system, and microcin) are located in a high-pathogenicity island (HPI), which we termed HPI-492 (158 kb). HPI-492 contains three transposons, named Tn*6497*, Tn*6498*, and Tn*6499* (Fig. 2). The other two virulence gene clusters (salmochelin and aerocin) and polysaccharide virulence genes (*rmpA*, *rmpA2*, and a truncated *rmpA2*) lie in the plasmid p11492-vir-CTXM. (Fig. 3). The resistance genes were further identified using ResFinder as *bla*_SHV-36_, *oqxA, oqxB*, and *fosA*, located on the chromosome, and *bla*_CTX-M-24_, located on the plasmid p11492-vir-CTXM.

Importantly, although most drug resistance and virulence genes are generally located in different plasmids (6), our analysis revealed a rare hybrid drug-resistant virulent plasmid, p11492-vir-CTXM (Table S5). It contains some known virulent genes (such as *rmpA* and *rmpA2*) and an extended-spectrum β-lactamase (ESBL) gene (*bla*_CTX-M-24_), contributing a lot to the MDR-hypervirulence phenotype. We then annotated p11492-vir-CTXM in detail. It was classified as a multireplicon IncHI1B/IncFIB plasmid using the PlasmidFinder database. We further performed a full-plasmid BLAST comparative analysis. The result showed that p11492-vir-CTXM exhibited 99% identity with the three IncHI1B/IncFIB virulent plasmids (pRJF999, GenBank accession no. CP014011; pK2044, GenBank accession no. AP006726; and pLVPK, GenBank accession no. AY378100), with high coverages (82%, 83%, and 76%, respectively) (Fig. 3A). Notably, p11492-vir-CTXM carried an ∼7-kb region harboring a *bla*_CTX-M-24_ gene that was unique compared with the other three virulent plasmids. If we excluded the unique region, the backbone region of p11492-vir-CTXM would show high similarity to those of pRJF999 and pK2044, with 99% identity and 99% coverage, which suggests that the rare hybrid plasmid is formed by the integration of the *bla*_CTX-M-24_ gene into the virulent plasmid.

We further explored the genetic basis for the integration of unique region into the virulence plasmid. This region is inserted into a conserved backbone gene encoding the permease of the drug/metabolite transporter (DMT), with two IS*903D* elements at terminal regions (in opposite directions). Each IS*903D* is in turn linked to an external 8-bp sequence (GCACAGAGA), possibly a product of target site duplications, indicating the insertion event of the *bla*_CTX-M-24_-embedded region. Additionally, this region is located in a novel transposon element, termed Tn*6501*, with the structure IS*903D-bla*_CTX-M-24_-IS*Ecp1*-ΔTn*1721*-ΔIS*Kpn19*-IS*903D*. Similar transposon genetic structure was also detected in the 68-kb plasmid pKP69 (GenBank accession no. EU195449.1) from Klebsiella pneumoniae, with 100% nucleotide identity and 84% coverage (Fig. 3B). These provided evidence of insertion of this region into the backbone of the virulence plasmid, based on IS*903D*-mediated intermolecular replicative transposition.

Although HvKP and MDR strains usually show nonoverlapping genotypes and phenotypes, our research demonstrated that a relatively infrequent MDR-HvKP strain evolved from HvKP through obtaining drug resistance genes. Furthermore, we discovered a rare hybrid plasmid, p11492-vir-CTXM, with both known virulent and *bla*_CTX-M-24_ genes_,_ although drug resistance and hypervirulence genes are unlikely within the same plasmid. The emergence of MDR-HvKP strains, especially that of those carrying drug-resistant virulent plasmids, poses unprecedented threats/challenges to public health. Mobile genetic elements may accelerate the formation of MDR-HvKP through horizontal gene transfer events. This is a dangerous tendency and should be closely monitored.

### Accession number(s).

Complete sequences of the chromosome of strain 11492 and of plasmid p11492-vir-CTXM have been deposited in the GenBank database under accession no. CP026021 and CP026022.

## Supplementary Material

Supplemental file 1

Supplemental file 2
