# Supplementary material for: Emergence of a Multidrug-Resistant Hypervirulent Klebsiella pneumoniae Sequence Type 23 Strain with a Rare blaCTX-M-24-Harboring Virulence Plasmid
Source: Antimicrob Agents Chemother. 2019 Feb 26;63(3):e02273-18. doi: 10.1128/AAC.02273-18 (PMC6395898; doi:10.1128/AAC.02273-18)
Supplement: Supplemental file 1 [file AAC.02273-18-s0001.pdf]

Supplemental Material

Emergence of a multidrug-resistant hypervirulent *Klebsiella pneumoniae* of ST23 with a rare *bla*$_{CTX-M-24}$-harboring virulence plasmid

Dingxia Shen[1,†], Guannan Ma[2,†], Cuidan Li[2,3,†], Xinmiao Jia[4,†], Chuan Qin[2,3,†], Tingting Yang[2,3], Lifeng Wang[1], Xiaoyuan Jiang[5], Nan Ding[2], Xiuli Zhang[2,3], Liya Yue[2], Zhe Yin[5], Lijun Zeng[5], Yongliang Zhao[6], Dongsheng Zhou[5,*], Fei Chen[2,3,*]

Table S1. Clinical information of the patient.

Case	Patient information
**Age**	> 30
**Hospital admission time**	November, 2014
**Source of hospital**	Chinese PLA General Hospital
**Diagnosis**	SAP, pancreatic abscess, fever
**WBC count (10^9/L)**	12.8
**Neutrophils (%)**	81.1
**LY (%)**	18.4
**CRP (mg/dL)**	18.2
**Temperature (°C)**	40.8
**Antibiotic therapy**	Imipenem-Cilastatin Sodium; Teicoplanin; Linezolid; Meropenem; Biapenem;
**Source of strain isolate**	blood

Abbreviations: PLA, People's Liberation Army; SAP, severe acute pancreatitis; WBC, white blood cell; LY, lymphocyte; CRP, C-reactive protein.

Table S2. Sequencing data of *K. pneumoniae* strain 11492 using SMRT sequencing platform.

Sample	11492
**Cell**	1
**Number of Bases (bp)**	384,396,596
**Mean Read Length (bp)**	8,762
**Mean Subread length (bp)**	7,543
**Coverage (X)**	61

Table S3. Sequencing data of *K. pneumoniae* strain 11492 using Hiseq sequencing platform.

Sample	11492
**Clean Reads**	9,548,040
**Duplication Deleted**	8,274,524 (86.66%)
**Filter**	8,203,572 (85.92%)
**MatePaired**	8,140,190 (85.26%)
**Mapped**	7,962,700 (83.4%)
**Unique Mapped**	7,874,315 (82.47%)
**Depth (×)**	218
**Cov10X**	98.96%

Table S4. Genome information of *K. pneumoniae* strain 11492.

Sample	Genome size(bp)	GC Content	Coding Genes	Average gene size (bp)	Coding region (bp)	tRNAs	rRNAs
**11492_chr**	5,252,687	57.45%	5,017	922	4,627,395 (88.10%)	87	25
**p11492-vir-CTXM**	193,176	50.37%	202	724	146,256 (75.71%)	0	0

Table S5. The detialed annotation of p11492-vir-CTXM (see the attached xls file).

Figure S1

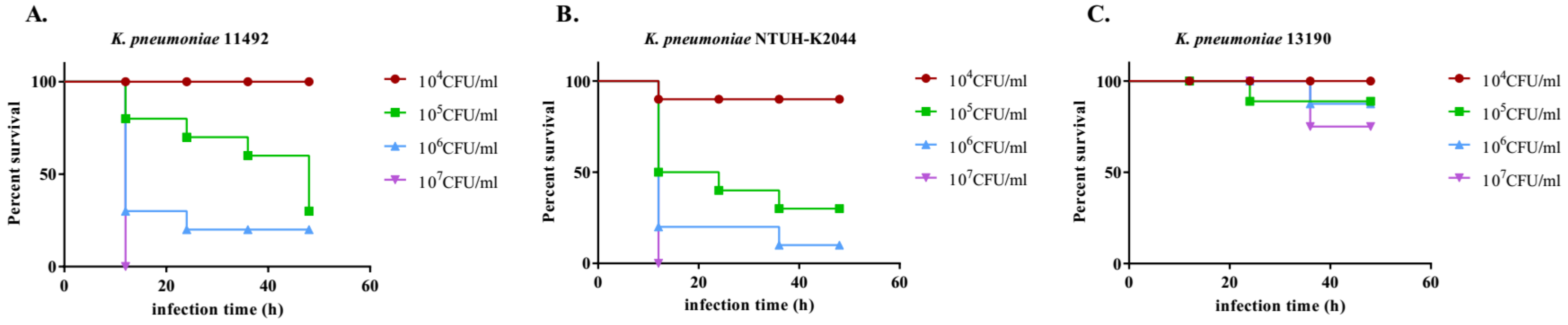

Figure S1. *G. mellonella* infection assessment of the three *K. pneumoniae* strains under various infection concentrations. Equal number of *G. mellonella* were infected with different concentrations of HvKP strain 11492 (A), reference HvKP strain NTUH-K2044 (B) and cKP strain 13190 (C). The virulence was determined by the survival rates at different infection timepoints.

Figure S2

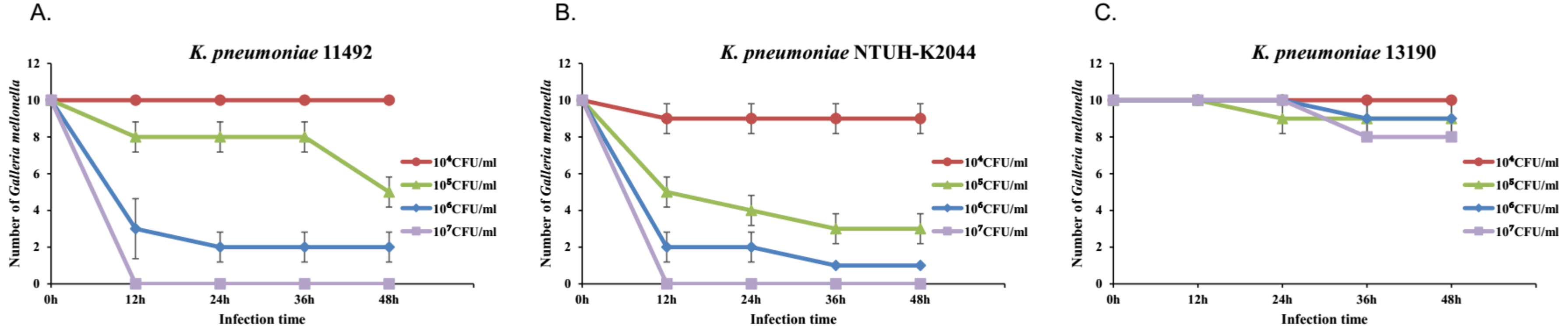

Figure S2. Survival curve of the *G. mellonella* infected with three *K. pneumoniae* strains under various infection concentrations. 10 *G. mellonella* were infected with the three *K. pneumoniae* strains under various concentrations. The virulence was determined by the survival numbers of *G. mellonella* when infecting with MDR-HvKP strain 11492 (A), reference HvKP strain NTUH-K2044 (B) and cKP strain 13190 (C) at different infection times. All data represent mean ± SD of three replicates.

Figure S3

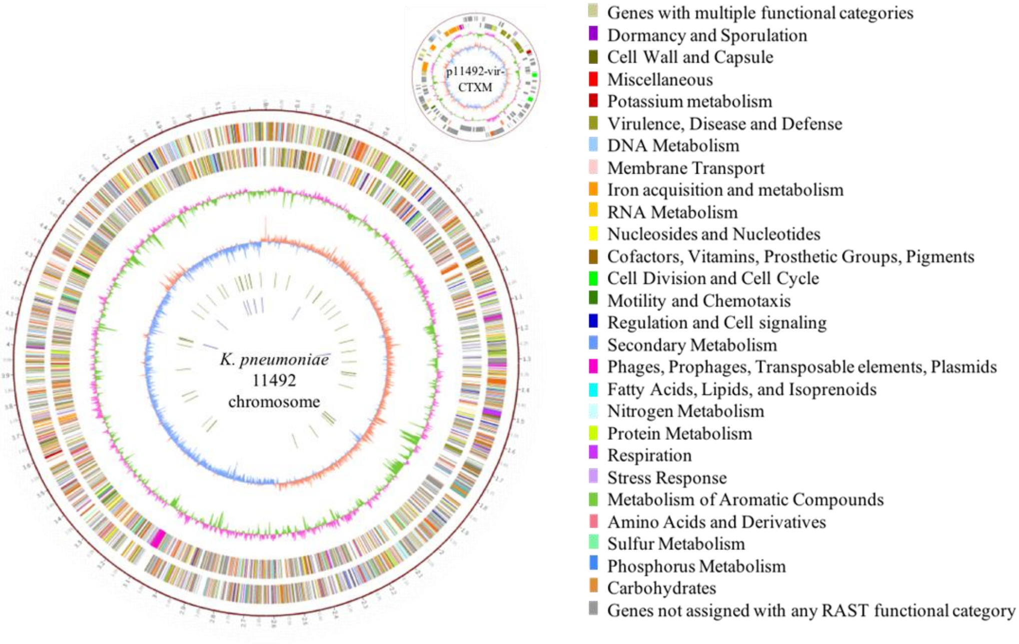

Figure S3. Circular representation of *K. pneumoniae* 11492 genome. Circles are shown as below (outside to inside) : (1) A physical map scaled in megabases (Mb) from base 1 (the start of the putative replication origin); (2) Coding sequences transcribed in a clockwise direction; (3) Coding sequences transcribed in a counterclockwise direction; (4) G+C content within a 2-kb sliding window (purple and green indicate G+C content that is higher and lower than average, respectively); (5) GC (G-C/G+C) skew within a 2-kb sliding window (red and blue indicate GC skew above and below zero, respectively); (6) tRNA genes; (7) rRNA genes. Genes displayed in 2 and 3 are color-coded according to different RAST functional categories shown on the right.
